# The prion protein family member Shadoo induces spontaneous ionic currents in cultured cells

**DOI:** 10.1038/srep36441

**Published:** 2016-11-07

**Authors:** Antal Nyeste, Claudia Stincardini, Petra Bencsura, Milica Cerovic, Emiliano Biasini, Ervin Welker

**Affiliations:** 1 Institute of Biochemistry, Biological Research Center, Hungarian Academy of Sciences, Szeged, Hungary; 2 Research Centre for Natural Sciences, Hungarian Academy of Sciences, Budapest, Hungary; 3 Dulbecco Telethon Laboratory of Prions and Amyloids, Center for Integrative Biology (CIBIO), University of Trento, 38123 Trento, ITALY; 4 Department of Neuroscience, Istituto di Ricerche Farmacologiche Mario Negri, 20156 Milano, ITALY

## Abstract

Some mutant forms of the cellular prion protein (PrP^C^) carrying artificial deletions or point mutations associated with familial human prion diseases are capable of inducing spontaneous ionic currents across the cell membrane, conferring hypersensitivity to certain antibiotics to a wide range of cultured cells and primary cerebellar granular neurons (CGNs). These effects are abrogated when the wild type (WT) form is co-expressed, suggesting that they might be related to a physiological activity of PrP^C^. Interestingly, the prion protein family member Shadoo (Sho) makes cells hypersensitive to the same antibiotics as mutant PrP-s, an effect that is diminished by the co-expression of WT-PrP. Here, we report that Sho engages in another mutant PrP-like activity: it spontaneously induces large ionic currents in cultured SH-SY5Y cells, as detected by whole-cell patch clamping. These currents are also decreased by the co-expression of WT-PrP. Furthermore, deletion of the N-terminal (RXXX)_8_ motif of Sho, mutation of the eight arginine residues of this motif to glutamines, or replacement of the hydrophobic domain by that of PrP, also diminish Sho-induced ionic currents. Our results suggest that the channel activity that is also characteristic to some pathogenic PrP mutants may be linked to a physiological function of Sho.

A great deal of evidence indicates that the prion protein (PrP^C^), encoded by the prnp gene, is involved in several neurodegenerative disorders, including Creutzfeldt-Jacob disease, Gerstmann-Sträussler-Scheinker syndrome, fatal familial insomnia, kuru, and possibly Alzheimer’s disease. These pathological conditions are characterized by protein aggregation and formation of β-sheet rich amyloid deposits, which might use PrP^C^ to deliver toxic signals^1^,^2^,^3^,^4^,^5^,^6^,^7^,^8^. However, the mechanism by which PrP^C^ transduces these signals is still not clear^9^,^10^,^11^. In this respect, a deeper understanding of the biology of the prion family proteins is essential for deciphering their complex interaction networks^12^,^13^ and for identifying those processes that are indeed involved in the above disorders.

Mutant forms of PrP carrying point mutations or deletions in the central region (CR, amino acid residues 105 through 125 in murine PrP) of the protein have provided a great deal of information regarding the potential toxic processes involving prion proteins^14^,^15^,^16^,^17^. A large part of data have been obtained with a mutant lacking the entire central region (referred to as ΔCR-PrP); transgenic mice expressing this mutant form [Tg(ΔCR)] on a prnp-null background exhibit neonatal lethality, massive spontaneous degeneration of cerebellar granule neurons (CGNs), and white matter pathology in the brain and spinal cord^18^. When Tg(ΔCR) mice are crossed with WT-PrP expressing mice, the phenotype is dose-dependently suppressed by the presence of the WT protein, expanding the lifespan from few weeks to more than 1 year when a five-fold excess is established. This suggests that WT-PrP and ΔCR-PrP act on the same molecular pathway, or else, that these two forms may physically interact^19^. Although the toxicity of ΔCR-PrP is not evident when using immortalized cells, Massignan *et al*. revealed that cells expressing this mutant form are hypersensitive to two classes of antibiotics, aminoglycosides and bleomycin analogues^20^. Interestingly, the co-expression of WT-PrP diminishes this hypersensitivity. Additional studies employing electrophysiology on ΔCR-PrP-expressing cells detected the presence of spontaneous large ionic currents in several kinds of immortalized cells^21^, primary neuronal cultures and cerebellar slices^22^. Again, the co-expression of WT-PrP silences these currents.

Further insights came from studies investigating two other members of the prion protein family^12^: doppel, a neurotoxic protein sharing structural similarities with the C-terminal globular fold of PrP^C^ 23, and Sho, a natively disordered protein displaying structural features similar to the N-terminal, flexible tail of PrP^C^ 24. Interestingly, the latter has been shown to possess WT-PrP-like protective properties, as its expression suppresses the toxicity of doppel and PrPΔ32-121, proteins that display ΔCR-PrP-like toxic effects^25^. Even more, when the hydrophobic domain (HD, amino acid residues 62 through 77 in murine Sho) - the only part of Sho bearing considerable sequence similarity with PrP^C^ - was deleted, these protective effects were no longer detectable. By contrast, when expressed in cultured immortalized cells (SH-SY5Y and HEK293) Sho expression caused hypersensitivity to Zeocin or G418, in a dose dependent manner, mimicking the expression of the ΔCR-PrP mutant. This phenotype was also rescued by the co-expression of WT-PrP^26^. All together, these data indicate that PrP^C^ and Sho may act on the same cellular pathways, via similar mechanisms.

In this study we describe a structural-functional characterization of the sequence determinants of Sho-induced cellular hypersensitivity to G418. Importantly, we also report that, similarly to ΔCR-PrP, Sho spontaneously induces ionic currents across the plasma membrane, as detected by patch clamping experiments.

## Results

### The role of the (RXXX)_8_ motif in the drug sensitizing activity of Sho

**Figure 1:**
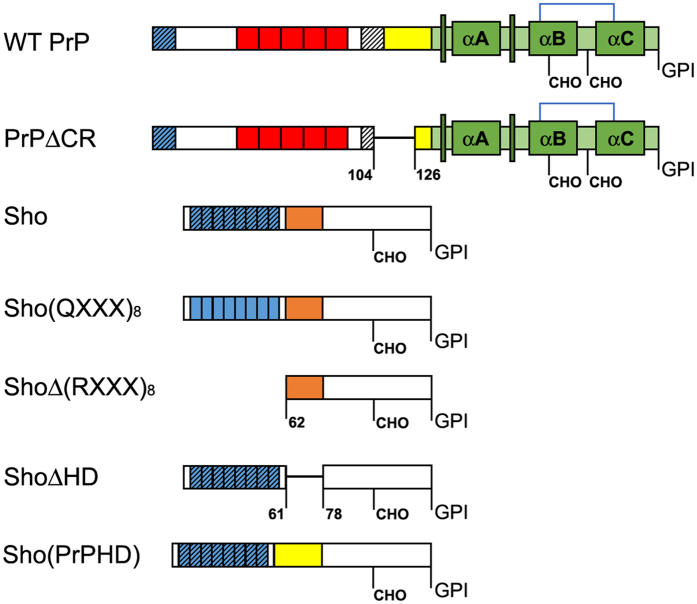
PrP and Sho constructs used in the studies. Schemes of the polypeptide chains indicate the domain structures and features of interest. αA, αB, αC: alpha helices. Narrow, tall, dark green boxes: β-strands. CHO: N-glycosylation sites. GPI: GPI-anchors. Blue brackets: disulphide bonds. Striated boxes: polybasic regions (PB1 (blue), PB2 (white) in PrP, (RXXX)_8_ in Sho), whereas, the non-striated blue boxes in Sho(QXXX)8 construct indicate the (QXXX)_8_ region. Red boxes in PrP: octarepeat region (OR). Yellow and orange boxes: hydrophobic domains (HD) of PrP or Shadoo, respectively. Numbers indicate the last and first residues neighbouring the deletions. Green tones: structured region of PrP.

**Figure 2:**
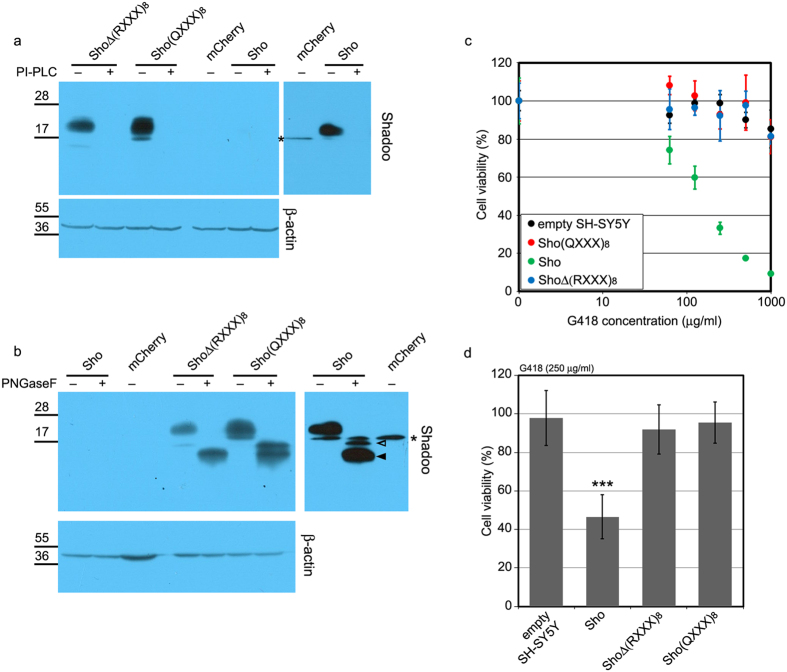
The presence of the (RXXX)_8_ domain is necessary for the drug sensitizing activity of the Sho protein. (**a**) Western blot analysis of the (RXXX)_8_ domain mutant Sho constructs’ cell surface localization. Cell lysates from mCherry, Sho, ShoΔ(RXXX)_8_ and Sho(QXXX)_8_ expressing SH-SY5Y cells, incubated with (+) or without (−) PI-PLC are tested (upper left panel). On the upper right panel lanes of mCherry and Sho expressing SH-SY5Y cells of the same membrane after longer exposure are shown. β-actin was used as loading control (lower panel). Immunoblotting of the respective medium supernatants for the presence of Sho is presented in Supplementary Fig. S2. (**b**) Western blot analysis of (RXXX)_8_ domain mutant Sho constructs’ N-glycosylation in lysates of mCherry, Sho, ShoΔ(RXXX)_8_ and Sho(QXXX)_8_ expressing SH-SY5Y cells, treated (+) or not treated (−) by PNGaseF. On the upper right panel lanes of mCherry and Sho expressing SH-SY5Y cells of the same membrane after longer exposure are shown. Empty and filled arrowheads mark the full length Shadoo and the C1 fragment, respectively. β-actin was used as loading control (lower panel). (**a,b**) *: marks nonspecific bands. Numbers and marks on the left indicate the positions of the corresponding molecular-weight size markers in kDa. Cropped images of immunoblots. Full length blots are presented in Supplementary Fig. S4. (**c,d**) Δ(RXXX)_8_ or (QXXX)_8_ mutations interfere with G418 hypersensitivity caused by the WT-Sho in SH-SY5Y cells (48 hours treatment). 100% is the fluorescence value of untreated control of each cell line. (**c**) Cytotoxicity assay, using PrestoBlue reagent: representative experiment carried out at G418 concentrations between 0 and 1000 μg/ml on various cells, as indicated. Values are means ± S.D. of corresponding replicas within the experiment. (**d**) Bars show the means ± S.D. of cell viabilities measured at 250 μg/ml G418 concentration in n = 3 independent experiments. Samples were compared to non-transfected SH-SY5Y cells, ***p < 0.001.

In order to display a toxic effect, ΔCR-PrP requires an intact PB1 domain (amino acid residues 23 through 27), a positively charged short segment^17^,^27^,^28^,^29^. This domain is also essential for the protective activities of WT-PrP^30^. In order to examine whether the N-terminal domain of Sho [(RXXX)_8_ motif^31^] is also essential for its drug-sensitizing activity^26^, we engineered a mutant Sho protein construct lacking amino acids 25 through 61 [ShoΔ(RXXX)_8_] (Fig. 1). Using an expression system employed previously for the expression of WT-Sho in SH-SY5Y cells, which harbour no detectable amount of endogenous Sho^26^, we developed cells stably expressing this deletion-mutant. Both WT and mutant Sho correctly localize at the cell surface, being attached to the plasma membrane via glycosylphosphatidylinositol (GPI) anchors (Fig. 2a and Supplementary Fig. S2) and are N-glycosylated (Fig. 2b). The mobility shift caused by the deletion of the (RXXX)_8_ motif is detectable when the protein is deglycosylated (Fig. 2b) as a result of peptide N-glycosidase F (PNGaseF) treatment. The expression level of the Δ(RXXX)_8_ mutant Sho protein in the cells is higher than that of the WT-Sho (Fig. 2a and Supplementary Fig. S3). The presence of the WT-Sho protein causes SH-SY5Y cells to be hypersensitive to G418 treatment i.e.: a considerable decrease in the viability of the cells is apparent at drug concentrations where the control SH-SY5Y cells seem to be unaffected. Despite the higher abundance, the presence of the Δ(RXXX)_8_ mutant does not confer a drug-sensitizing phenotype to the cells. To see if the positively-charged amino acids are indeed the critical determinants of the toxicity-associated Sho phenotype, we mutated all eight arginines of the (RXXX)_8_ motif to glutamines (Fig. 1). This mutant protein [Sho(QXXX)_8_] is processed by the cells like WT-Sho, as assessed by phosphatidylinositol-dependent phospholipase C (PI-PLC) and PNGaseF treatment combined with western blot, and reaches expression levels higher than that of WT-Sho (Fig. 2a,b, and Supplementary Fig. S3). Nevertheless, no significant differences are discernible when these cells are compared to the control SH-SY5Y, or to the deletion mutant [ShoΔ(RXXX)_8_] protein expressing ones in a G418 viability assay (see Fig. 2c,d). These results indicate a critical role for the (RXXX)_8_ motif of Sho in the observed drug hypersensitivity phenotype.

### The role of the hydrophobic domain in the drug sensitizing activity of Sho

**Figure 3:**
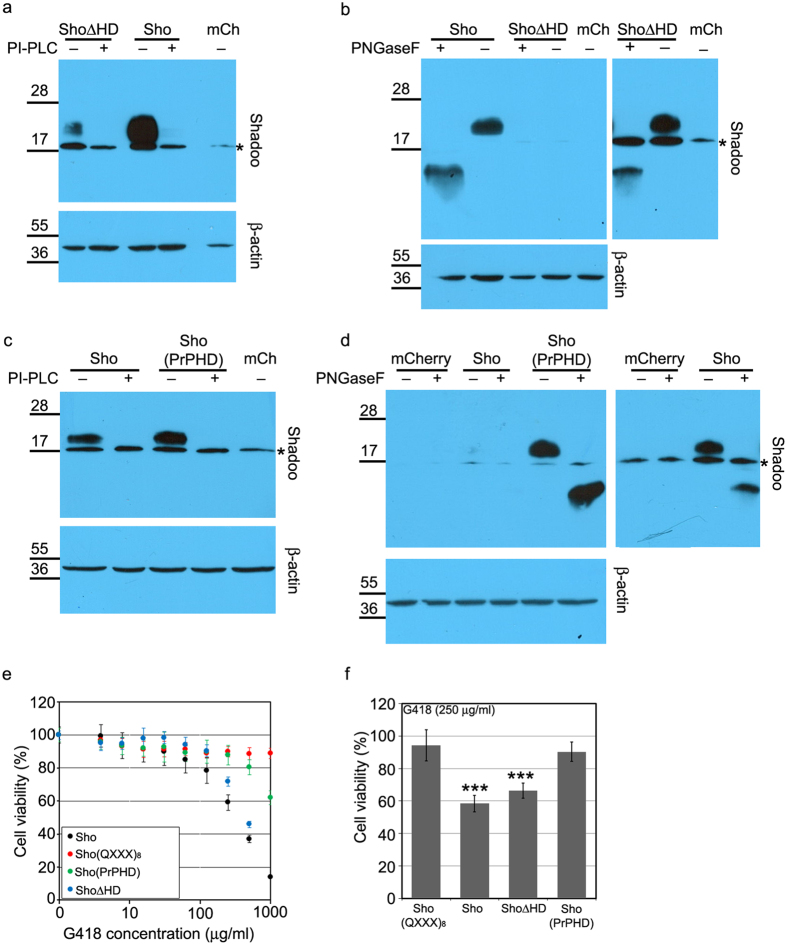
The hydrophobic domain (HD) of the Sho protein is not necessary for the drug sensitizing activity but modulates it. (**a**) Western blot analysis of ΔHD mutant Sho construct’s cell surface localization. Cell lysates from mCherry, Sho and ShoΔHD expressing SH-SY5Y cells, incubated with (+) or without (−) PI-PLC are tested (upper panel). (**b**) Western blot analysis of Sho N-glycosylation in lysates of the indicated cells, treated (+) or not treated (−) with PNGaseF (upper left panel). On the upper right panel lanes of mCherry and ShoΔHD expressing SH-SY5Y cells of the same membrane after longer exposure are shown. (**c**) Western blot analysis of Sho(PrPHD) construct’s cell surface localization. Cell lysates from mCherry, Sho and Sho(PrPHD) expressing SH-SY5Y cells, incubated with (+) or without (−) PI-PLC are tested (upper panel). (**d**) Western blot analysis of Sho N-glycosylation in lysates of the indicated cells, treated (+) or not treated (−) with PNGaseF (upper left panel). On the upper right panel lanes of mCherry and Sho expressing SH-SY5Y cells of the same membrane after longer exposure are shown. (**a–d**) *Marks a nonspecific band. Numbers and marks on the left indicate the positions of the corresponding molecular size markers in kDa. An intracellular protein, β-actin was used as loading control for cell lysates (lower panels). Cropped images of immunoblots. Full length blots are presented in Supplementary Fig. S4. (**e,f**) Deletion of HD (ShoΔHD) doesn’t interfere with G418 hypersensitivity caused by Sho in SH-SY5Y cells (48 hours treatment), but replacement of Sho HD with PrP HD [Sho(PrPHD)] reduces this effect. 100% is the fluorescence value of untreated control of each cell line. Sho(QXXX)_8_ construct was used as non-sensitizing negative control. (**e**) Cytotoxicity assay, using PrestoBlue reagent: representative experiment carried out at G418 concentrations between 0 and 1000 μg/ml on various cells, as indicated. Values are means ± S.D. of corresponding replicas within the experiment. (**f**) Bars show the means ± S.D. of cell viabilities measured at 250 μg/ml G418 concentration in n = 3 independent experiments. Samples were compared to SH/Sho(QXXX)_8_ cells, ***p < 0.001.

The hydrophobic domains, the part of PrP^C^ and Sho that share more extended sequence similarity, have considerable influence on the toxic/protective phenotypes of PrP^C^ 32 and Sho^33^. To assess its role in the drug hypersensitising phenotypes conferred by Sho expression, we deleted Sho’s HD (amino acid residues 62 through 77; ShoΔHD, Fig. 1) and expressed the mutant protein in SH-SY5Y cells. Figure 3a,b shows that ShoΔHD is on the cell surface and is N-glycosylated, respectively, although its expression reaches much lower level than that of the WT protein (Fig. 3a, and Supplementary Fig. S3). Since the epitope of the anti Sho antibody used in this experiment lies outside of the deleted region, it is unlikely that the weaker signals of ShoΔHD on the blot are related to an altered affinity of the antibody to the mutant Sho protein. The deletion of HD does not diminish the ability of Sho to cause G418 hypersensitivity to cells (Fig. 3e,f). Considering the much lower expression level of ShoΔHD (at a similar level WT-Sho exhibits only reduced activity^26^), it is likely that ShoΔHD is more effective to induce this phenotype than WT-Sho. While these experiments suggest that the presence of Sho’s HD is not required for conferring drug hypersensitivity, its absence seems to aggravate it.

To clarify the importance of this region of PrP in the drug hypersensitivity phenotype, we replaced the HD of Sho with that of PrP^C^ [amino acid residues 111 through 133; Sho(PrPHD), Fig. 1]. Figure 3c,d shows that this mutant also localizes on the cell surface and is glycosylated in SH-SY5Y, respectively. Even though the expression level of this mutant was higher than that of WT-Sho, the presence of the HD segment of PrP^C^ greatly suppressed the drug-sensitizing effect of Sho, although, at higher G418 concentrations the effect was still detectable (Fig. 3e,f).

### Sho causes spontaneous ionic currents in two different cell lines

**Figure 4:**
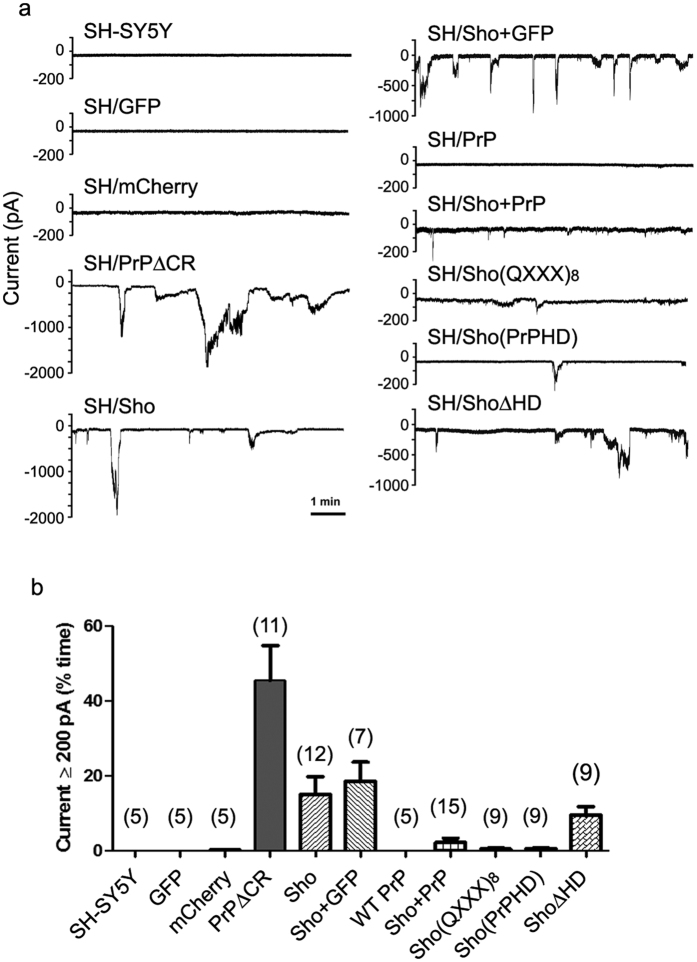
Sho protein, akin to PrPΔCR induces inward ionic currents. (**a**) Representative traces of whole cell patch clamp recordings on SH-SY5Y cells non-transfected or expressing the indicated proteins: wild-type or mutant Sho or PrP, or the empty vectors (possessing EGFP or mCherry expression reporter) at holding potential of −80 mV. (**b**) Current activity recorded in the different types of SH-SY5Y cell clones, plotted as percentage of total time the cells exhibited inward currents ≥200 pA. (Mean ± SEM; SH-SY5Y: no currents detected; GFP: no currents detected; mCherry: 0.3 ± 0.1; PrP∆CR: 45.4 ± 9.4; Sho + mCherry: 14.9 ± 4.9; Sho + GFP: 18.5 ± 0.1; PrP: no currents detected; Sho + PrP: 2.0 ± 0.01; 307: 0.46 ± 0.01; 309–250: 0.54 ± 0.0; 324–200: 11.67 ± 0.05). In brackets the number of samples for each cell type is indicated. Of note, in approximately 25% of PrP∆CR or Sho cells, recordings lasted less than 2 min. These data, which may reflect an intrinsic cell fragility, were omitted from the analyses.

**Figure 5:**
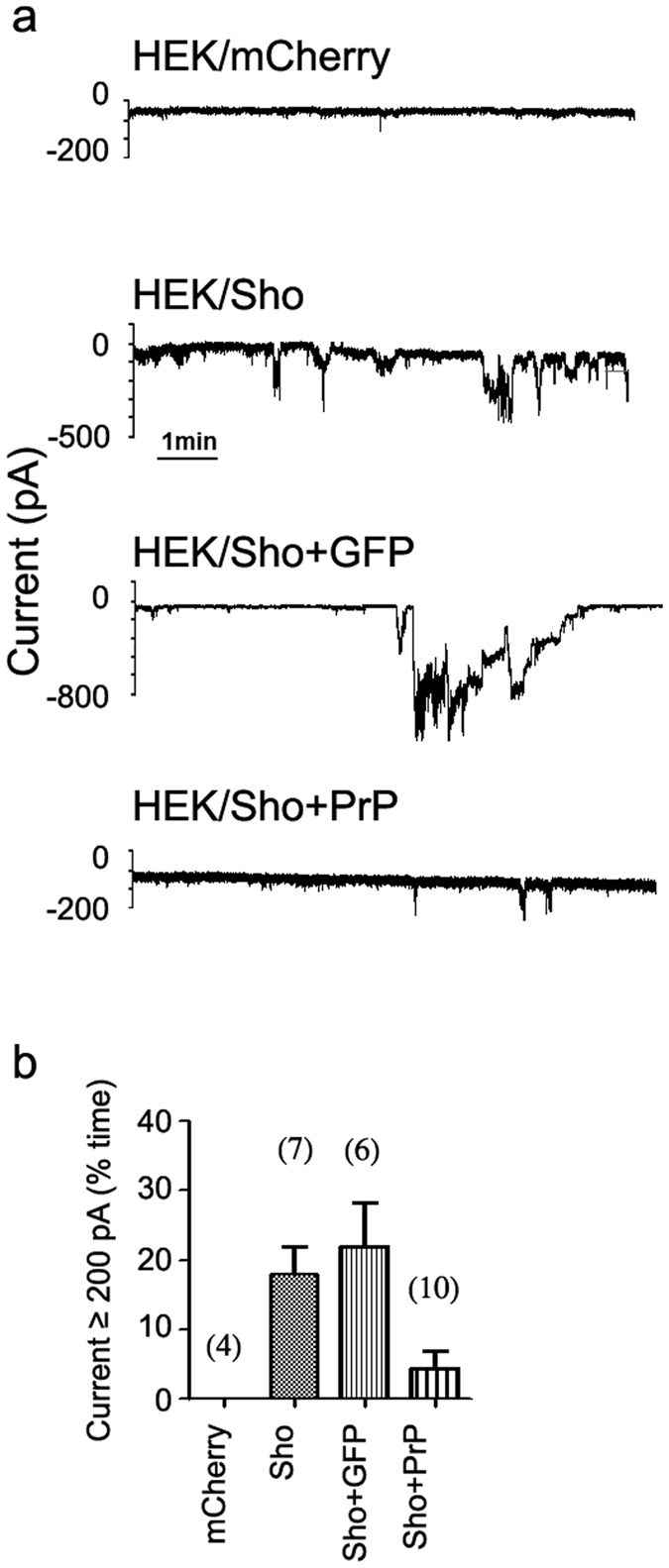
Sho protein expressed in HEK293 cell line induces inward ionic currents. (**a**) Representative traces of whole cell patch clamp recordings on HEK293 cells expressing mCherry only or the wild-type Sho, as well as co-expressing Sho and GFP or PrP at holding potential of −80 mV. (**b**) Current activity recorded in the different types of HEK293 cells, plotted as percentage of total time the cells exhibited inward currents ≥200 pA. (Mean ± SEM; mCherry: no currents detected; Sho: 17.8 ± 4.1; Sho + GFP: 22.0 ± 6.4; Sho + PrP: 4.4 ± 2.4). The number of recorded cells for each type is indicated in brackets.

It was previously reported that ΔCR-PrP, as well as other mutant PrP forms carrying deletions or disease-associated point mutations in the central region, can induce spontaneous ionic currents in cultured cells and primary neurons^21^. Similarly to the drug-hypersensitizing effect, these currents are abrogated by the co-expression of WT-PrP. To assess whether Sho can also induce similar currents, we carried out patch clamp experiments on SH-SY5Y cells expressing either WT-Sho or different Sho mutants (Fig. 4). We found that expression of Sho induces ΔCR-PrP-like ionic currents, even though the size, frequency and duration of these bursts are generally of lower magnitude as compared to ΔCR-PrP (Fig. 4a). As a possible result of the currents, we also detected a significant alteration in resting membrane potentials in Sho-expressing cells, as compared to controls [the measured resting membrane potentials were as follow: non-transfected SH-SY5Y: 44 ± 3 mV; GFP-expressing cells: 38 ± 4 mV; mCherry-expressing cells: 37 ± 5 mV; PrP ∆CR-expressing cells: 24 ± 2 mV; Sho-expressing cells: 28 ± 3 mV; Sho + GFP-expressing cells: 28 ± 4; PrP WT-expressing cells: 39 ± 5 mV; Sho + PrP-expressing cells: 41 ± 4 mV; Sho(QXXX)_8_-expressing cells: 40 ± 6 mV; Sho(PrPHD)-expressing cells: 38 ± 4 mV; Sho ∆H-expressing cells: 30 ± 3 mV]. Importantly, we also observed that co-expression of WT-PrP silences Sho-associated currents. Both Sho-induced currents and the PrP’s silencing activity were observed also in HEK293 cells (Fig. 5). This observation indicates that, as reported previously for ΔCR-PrP, both effects apparently occur independent of the cell line in which the proteins are expressed. Moreover, mutation of the arginine residues within the (RXXX)_8_ motif to glutamines abolishes the current-inducing abilities of Sho, while deletion of the HD had no such effects. Interestingly, replacement of the HD of Sho with that of PrP silenced these Sho-induced currents (Fig. 4b). Collectively, these results demonstrate that Sho is capable of inducing spontaneous ionic currents at the cell membrane, mimicking the channel activity of toxic PrP mutants.

## Discussion

The most significant finding of these studies is that Sho expression spontaneously generates ionic currents similar to those induced by ΔCR-PrP and other disease-associated PrP mutants.

Several evidences suggest that the nature of the Sho-induced currents is similar or even identical to that induced by ΔCR-PrP. Both currents are highly irregular, fluctuating randomly over a time course of seconds to minutes, and reaching amplitudes of several thousand pA. Furthermore, co-expression of WT-PrP silences the currents in both cases. In addition, the replacement of the HD in the sequence of Sho with the HD of the prion protein also diminishes these currents, suggesting that identical roles are played or undertaken by PrP HD in case of both proteins and both processes. Another parallel between Sho and mutant PrP-s is their ability to confer drug hypersensitivity, as revealed by G418 treatment and viability assays. Consistent with the data collected with ΔCR-PrP, WT and mutant variants of Sho reveal a nearly perfect correlation between the induction of drug hypersensitivity and the channel activity, with some mutants either causing both ionic currents and drug hypersensitivity (Sho, ShoΔHD), or neither of them [ShoΔ(RXXX)_8_, and Sho(QXXX)_8_]. These results support the idea that the two phenotypes are intrinsically linked, with the drug hypersensitivity being a secondary effect of the inability of the cells to maintain ion homeostasis, affecting drug uptake^32^.

The molecular basis of the observed ionic currents is still not clear. The fact that Sho induces very similar currents to that of ΔCR-PrP challenges many of the previous interpretations.

It has been proposed that ΔCR-PrP molecules themselves may form cationic channels or pores in the cell membrane by the transient self-associations of PrP molecules^16^,^32^. WT-PrP molecules might also associate on the cell surface, however, without resulting in cationic permeable channels or pores. Previous studies speculated that one potential mechanism for this might be a transient electrostatic interaction between the N-terminal and the globular part^34^,^35^, that may physiologically be present in WT-PrP, and that is hindered from taking place in the absence of the CR (residues 105 through 125, also called hinge region), leaving the N-terminal free and able to penetrate the plasma membrane. The possibility that Sho would also be able to self-associate to form pores remains to be investigated.

Another interpretation is that Sho, like ΔCR-PrP may directly modulate the activities of specific ion channels either by interacting with them or by indirectly regulating their activity. While PrP^C^ has been suggested to influence the activity of several ion channels^36^,^37^ in case of Sho such an activity needs to be verified.

Our experiments might provide further insights into this process. We found that the deletion of the HD neither has an effect on ionic currents, nor diminishes the drug hypersensitizing ability of Sho. By contrast, inserting the HD of PrP^C^ to replace Sho’s HD diminishes both phenotypes. Since the presence of this domain is also required in WT-PrP to suppress the same activities, PrP HD seems to have a critical role in these processes, likely more than just functioning as a hinge and connecting the N- and C-terminus of PrP, as previously proposed^16^.

We found that the presence of the (RXXX)_8_ motif^31^ is essential for Sho to induce ionic currents or drug hypersensitivity; this could indicate either a direct or an indirect effect. Since the mutant Sho protein is correctly localised on the cell surface and altering only the positively charged character of the (RXXX)_8_ motif is sufficient to diminish the ionic currents and G418 hypersensitizing ability of Sho, it is more likely that the arginines of the (RXXX)_8_ motif themselves are involved in some interactions critical for this phenotype.

What is the nature of these interactions? One possible interpretation can be drawn from the analogy with the corresponding PrP region: The positively charged N-terminal KKRPK sequence (the first polybasic domain: PB1) was shown to function as a protein transduction domain, capable of aberrantly inserting the adjacent polypeptide segments through the lipid bilayer, giving rise to the observed currents^27^,^32^,^38^. In the frame of this scenario, Sho might form transient pores as well, and its positively charged N-terminus could possess similar transducing capabilities. Indeed, the (RXXX)_8_ motif resembles an α-helical amphipathic model peptide with transducing capacities: **K**LAL**K**LAL**K**AL**K**AAL**K**L^39^. Thus, although this conclusion still needs proof, it is tempting to speculate that the positively charged regions of PrP^C^ and Sho may share protein transducing characteristics, being able to penetrate and traverse the membrane, pulling a segment of the disordered polypeptide chains through the lipid bilayer. The hydrophobic domain of PrP^C^ might behave like transmembrane helices and may stabilize this transduced structure by positioning itself at a stable orientation across the membrane. The transduced polypeptide chains may or may not associate in the membrane by these regions, but in either case they result in no ionic currents. Being fully hydrophobic, even if they associate, HD-s cannot form membrane channels with polar residues facing inside that would allow exchange of polar molecules between the outside and the cell interior. The polybasic region between amino acid residues 100 and 109 (PB2, in murine PrP) may help to position the HD into the membrane bilayer; its deletion also induces currents^16^. In the absence of the HD the hydrophilic polypeptide segments of ΔCR-PrP molecules that are pulled into the lipid bilayer are forced to be stuck together to minimise contact with the hydrophobic core of the lipid bilayer and are disrupting the membrane integrity forming hydrophilic pores that are permeable for ions. The absence of transmembrane segments may render an unstable dynamic character to the transduced structure explaining the highly irregular nature of the currents. The drug hypersensitivity might be a secondary effect induced by the altered ion balance resulted from the formation of these pores. The Sho HD being shorter might not be able to stabilise itself in the membrane in a similar manner, thus, the protein may show the same phenotype as the deletion mutant PrP-s.

WT-PrP, when present, disrupts ΔCR-PrP and Sho channels. The rescue by WT-PrP calls for additional details to be added to this model, such as the interaction of WT-PrP with mutant prion proteins, and with Sho. In support of this model, a direct *in vivo* (and *in vitro*) interaction between PrP^C^ and Sho has been suggested by a few studies^13^,^40^. However, these associations cannot be mediated by an interaction between the HD-s^41^, since the HD is not present in some toxic mutant variants.

Obviously, other proteins could contribute to the formation of mutant PrP- and Sho-induced channels. The effects found with point mutations in the central region of PrP^21^ present additional challenge to this model. The mutations (P101L, G113V, and G130V) do not change much the hydrophobic character of the region but still induce channel activity and drug hypersensitivity, indicating that the HD needs additional structural requirements than just the hydrophobic character for stabilising a structure where the transduced polypeptides are merged in the lipid bilayer and positioned with their hydrophobic segments.

In the alternative model, PrP interacts with an ion channel complex in a dual mode: binding of the C-terminal globular domain of PrP to the complex is potentiated by the polybasic region of PrP (PB1). This binding causes dysfunction of the channel showing unregulated opening and triggering the detected toxic signals, an effect that is attenuated by the binding of the N-terminal parts involving the HD to a second site. In the lack of the first polybasic region, the C-terminal domain does not bind to the complex. Deletion or alteration of the HD blocks its proper binding to the second site of the channel complex resulting in spontaneous ionic currents. Depending on the structural alteration caused by each mutation, PrP mutants may show decreased ability to induce ionic currents in parallel with a less severe *in vivo* phenotype. WT-PrP would compete for both binding sites, rescuing the detrimental effects of each mutant. It can be envisaged that Sho also interacts with such complex to induce spontaneous currents. However, since, no interaction of Sho with ion channels has been reported so far, this possibility remains to be proven.

Prion protein exhibits dual activity, with the WT protein displaying protective phenotypes while some mutant variants with internal deletions are being involved in toxic processes^2^. Interestingly, in case of Sho the WT protein is the one that itself exerts either protective or toxic activities depending on the experimental paradigms examined^25^,^26^,^40^. However, none of the phenotypes of Sho-expressing cells seen *in vitro*, protective or toxic, are apparent *in vivo*. One would expect that if the protective activity of Sho seen *in vitro* prevails *in vivo* this effect should be apparent in Tg(ΔCR) mice where the presence of WT-PrP does not mask this protective activity. Although, in lack of universal anti-Sho antibodies it might not be trivial to gain clear distinction between expressing and non-expressing tissues; using a combinations of three anti-Sho antibodies it has been argued that the reason why Sho does not manifest a protective role in Tg(ΔCR) mice is due to a lack of its expression in CGNs^25^. PrP^C^ has been shown to be able to perform its protective activities in trans^42^,^43^. It would be also interesting to know if Sho can exert its protective effects in trans, and, if the glial cells of the region have any Sho expression, which should provide protection to Tg(ΔCR) mice CGNs in the lack of Sho expression in CGNs themselves. By contrast, if *in vivo* Sho is involved in toxic activities, such as observed *in vitro*, this should be apparent in prnp-null mice where the co-expression of the endogenous PrP does not diminish this effect. In the absence of such effects that are apparent in the Shmerling^14^ and Tg(ΔCR)^18^ mutant mice, it is also speculated that the endogenous Sho expression level might be much lower than that of PrP, that is simply too low for displaying a toxic effect in prnp-null mice^26^. Crossing of Sho over-expressing mice with prnp-null or Tg(ΔCR) mice could clarify the second as well as the first issue, respectively.

## Methods

### Chemicals, reagents, antibodies

Restriction endonucleases, T4 DNA ligase and Pfu DNA polymerase were purchased from Thermo Fisher Scientific. DNA oligonucleotides were from Microsynth AG (http://microsynth.ch). High-glucose Dulbecco’s Modified Eagle Medium (DMEM) and fetal bovine serum (FBS) were obtained from Thermo Fisher Scientific (Gibco) and Penicillin/Streptomycin from Lonza. Proteinase inhibitor cocktail, Calpain inhibitor I, G418 were obtained from Sigma-Aldrich. Bradford-reagent was from Bio-Rad. Polyvinylidene fluoride (PVDF) transfer membrane and chemiluminescent substrate (Immobilon ECL substrate) were from Millipore. PNGaseF was purchased from New England Biolabs. Phosphatidylinositol dependent phospholipase C (PI-PLC), and PrestoBlue reagent were obtained from Thermo Fisher Scientific. The following primary antibodies were used: anti-Sho rabbit polyclonal antibody (Abgent, AP4754b), anti-β-actin mouse monoclonal antibody (Sigma, A1978). Secondary antibodies used were: Horseradish peroxidase (HRP)-conjugated anti-mouse and anti-rabbit IgG were from Jackson ImmunoResearch (715-035-151) and Pierce Biotechnology (31460), respectively. All other reagents and chemicals were purchased from Sigma-Aldrich.

### Plasmid constructs and DNA cloning

The Sleeping Beauty and pRRL vectors containing EGFP, PrP, PrPΔCR, mCherry, or Sho expression cassettes (see Table 1) were prepared earlier and are described in ref. 26.

Preparation of LV/ShoΔ^(RXXX)8^(R) and LV/Sho^(QXXX)8^(R) plasmid vectors were carried out briefly, as follows. The plasmids encoding either ShoΔ25–61 [ShoΔ(RXXX)_8_] or Sho(QXXX)_8_ were prepared earlier and are described in ref. 31. The DNA fragments harbouring the Δ(RXXX)_8_ and (QXXX)_8_ mutations in Sho cDNA were cut from the respective plasmids by NdeI and Eco81I restriction enzymes and were inserted between the same restriction sites of the LV/Sho(R) plasmid, replacing WT Sho cDNA with the deletion mutant constructs.

Preparation of LV/ShoΔ^HD^(R) plasmid vector was carried out briefly, as follows. The Sho HD (amino acid residues 62 through 71) was deleted by using QuikChange site directed mutagenesis and the following mutagenesis primers: *Sho*Δ*HD-fwd* and *Sho*Δ*HD-rev*. The DNA fragment encoding a CMV-IE promoter followed by the ShoΔHD cDNA was amplified by PCR with the following PCR primers: *V*-Δ*HD-fwd* and *V*-Δ*HD-rev*. The amplified DNA product containing ShoΔHD coding sequence was digested and inserted between the NdeI and SapI sites of the LV/Sho(R) vector, replacing WT Sho cDNA.

Preparation of LV/Sho^(PrPHD)^(R) plasmid vector. The HD in Sho cDNA was replaced by the HD of PrP briefly, as follows. The DNA linker (oligos PrPHDlin-fwd and PrPHDlin-rev) encoding mPrP HD (amino acid residues 111 through 133) was ligated between two PCR products, one harbouring a CMV-promoter followed by a Kozak-sequence and an 5′ fragment of the mSho cDNA (encoding amino acid residues 1 through 61) and another fragment harbouring a 3′ fragment of the mSho cDNA (encoding amino acid residues 78 through 147). The PCR products encoding the N and C-terminal Sho fragments were amplified from the LV/Sho(R) vector using the following primers: *V-CMV-PrP-fwd* and *Sho-HD5-rev* (N-terminal fragment) and *Sho-HD3-fwd* + *V-ins-rev* (C-terminal fragment) and were digested by the restriction enzyme BsmBI, producing a 5′TCGC overhang on the 3′ side of the 5′-fragment and a 5′GGCC overhang on the 5′ end of the 3′-fragment. The ligation of the two fragments and the linker was followed by the PCR amplification of the full DNA sequence encoding the chimeric protein Sho(1–61)-PrP(111–133)-Sho(78–147) [will be called further as Sho(PrPHD)] with the following primers: *CMV-in-fwd* and *V-CMV-PrP-rev*. The amplified DNA fragment encoding the chimeric Sho(PrPHD) was digested and inserted between NdeI and ApaI restriction sites of the LV/Sho(R) plasmid, replacing WT Sho cDNA.

Sequence correctness for the expression cassettes in all plasmids generated in this study was confirmed by Sanger sequencing (Microsynth AG).

Figure 1 summarizes the constructs (PrP and Sho) used in this study. DNA oligonucleotide sequences used for cloning are available in Supplementary Table S1.

### Cell lines, culturing, transfection and transduction

The maintenance, transfection or transduction of SH-SY5Y human neuroblastoma cell line (ATCC CRL-2266^TM^) was carried out as described in ref. 26. The Shadoo-expressing and control transgenic HEK293 cell lines used in this study (Table 1) were established earlier and are described in ref. 26. The lentiviruses were generated in the Hungarian National Blood Transfusion Service’s lentiviral facility.

All types of cells were regularly tested for mycoplasma contamination. EGFP and mCherry positivity were examined at every passage and experiments were carried out on cultures in which at least 90% of the cells expressed the required fluorescent markers. In parallel with the execution of the experiments, the expression levels of the transgenes were determined by immunoblotting technique. Table 1 lists the cell types used in this study.

### Immunoblotting

Cells seeded on 100 mm cell culture dishes were harvested at 70–90% confluence by washing once with PBS and scraping in 1 ml PBS. Harvested cells were pelleted by centrifugation (3 min, 200 × g) and re-suspended in ice cold lysis-buffer (50 mM HEPES, pH 7.5, 0.2 mM EDTA, 10 mM NaF, 250 mM NaCl, 0.5% NP-40, with 1% Proteinase inhibitor cocktail, 1% Calpain inhibitor, 1 mM DTT). The total protein concentration was measured by using Bradford protein assay. Where needed, PNGaseF treatment was carried out on samples of 20 μg total protein, according to the manufacturer’s protocol. Samples of 20 μg total protein, depending on the necessities of the experiment, were run on 15% denaturing polyacrylamide gels and were blotted onto activated PVDF membrane, using a wet blotting system from Bio-Rad. The membrane was blocked for at least 1 hour in Tris buffered saline with Tween 20 (TBST), containing 5% nonfat milk powder, and primary antibodies were applied overnight, at 4 °C at the following dilutions: anti-Sho rabbit polyclonal antibody 1:200, anti-β-actin mouse monoclonal antibody 1:4000. The next day, excess primary antibodies were removed by several washing steps in TBST and HRP-conjugated secondary antibodies were applied to the membrane for 60 minutes, room temperature, at the following dilutions: anti-mouse 1:20000, anti-rabbit antibody 1:200000, in blocking buffer. The proteins were visualized on X-ray film after adding chemiluminescent substrate (Millipore Immobilon ECL substrate) to the membrane. Uncropped western blot photos are available online in Supplementary Data. Densitometry analyses were carried out on at least 3 independent western blots, using ImageJ 1.48v software. For densitometry purposes non-saturated bands were chosen. When different proteins could not be detected on the same film due to their different expression levels (i.e. Fig. 2a), different exposition times of the same membrane were used and the corresponding measured band densities below saturation were corrected for the ratio of the two exposition times as a rough estimate for comparison.

### Phosphatidylinositol-dependent phospholipase-C (PI-PLC) treatment

Cells were seeded on 24-well plates. After reaching confluence, the PI-PLC treatment was carried out according to the manufacturer’s protocol. Briefly: cells were washed twice in PBS and the plate with cells having only PBS or PBS with PI-PLC (1 unit/ml PI-PLC) was rocked gently for 30 minutes at 4 °C. The supernatant media were removed from the cells, and the PI-PLC treated and untreated cells were harvested from the plates by scraping and were processed for SDS-PAGE. Detection of Shadoo in the media was carried out, as follows: media of PI-PLC treated and untreated conditions were removed and supplemented with 1% Proteinase inhibitor cocktail, 1% Calpain inhibitor, 1 mM DTT. Total protein in the media samples was precipitated with trichloroacetic acid treatment (~6% final TCA concentration, 30 minutes on ice, followed by a 30 minute centrifugation at 9000 × g, 4 °C). The precipitated protein was resuspended in 1× loading dye and 20 μl for each was loaded on gels.

### Cell viability assays

Cells stably transfected or transduced were seeded onto 96-well plates at 3 * 10^4^ cells/well density (SH-SY5Y cells). After the attachment of the cells, cells were treated by G418 by changing the medium to fresh containing serial dilutions of G418 and culturing the cells for additional 48 hours. After G418 treatment, the medium was replaced by PBS containing 5% PrestoBlue and cells were placed back into the CO_2_ incubator for additional 60 minutes before measuring fluorescence with a Perkin Elmer Viktor X3 2030 Multilabel Reader (excitation: 555 nm, emission: 585 nm).

### Whole cell patch clamping

Whole cell recordings were performed using borosilicate patch pipettes (3–5 MΩ). Cells were visualized with 40X objective using an Olympus BX51WI microscope equipped with reflected fluorescence as well as differential interference contrast observation systems. Experiments were conducted at room temperature with the following solutions. Internal: 140 mM Cs-glucuronate, 5 mM CsCl, 4 mM MgATP, 1 mM Na2GTP, 10 mM EGTA, and 10 mM HEPES (pH 7.4 with CsOH); External: 150 mM NaCl, 4 mM KCl, 2 mM CaCl_2_, 2 mM MgCl_2_, 10 mM glucose, and 10 mM HEPES (pH 7.4 with NaOH). Data were acquired using a Multiclamp 700B amplifier and pClamp 10 software (Molecular Devices, Foster City, CA), and sampled at 5 kHz with a Digidata 1440 (Molecular Devices, Foster City, CA).

### Statistics

Cell viability assays were done with 5 parallel samples for every condition. The number of surviving cells in case of each drug concentration was normalized to the number of cells receiving no drug treatments. For statistical analysis, concentration of 250 μg/ml G418 was chosen.

Statistical analysis (Normality tests, Student’s t-tests, One-way ANOVAs with two-tailed Dunnett’s or Tukey’s HSD post-hoc tests) was carried out on data from at least 3 independent experiments with SPSS Statistics v20 software. On plots, mean ± standard deviation (SD) values are shown. p values: *0.01 < p < 0.05, **0.001 < p < 0.01, ***p < 0.001.

## Additional Information

**How to cite this article**: Nyeste, A. *et al*. The prion protein family member Shadoo induces spontaneous ionic currents in cultured cells. *Sci. Rep*. **6**, 36441; doi: 10.1038/srep36441 (2016).

**Publisher’s note**: Springer Nature remains neutral with regard to jurisdictional claims in published maps and institutional affiliations.

## Supplementary Material

Supplementary Information

## Figures and Tables

**Table 1:** Cells with stable transgene expression used in these studies.

Name of transgenic cells	Introduced transgenes	Parental cell	Vector used
SH/GFP*	EGFP	SH-SY5Y	SB/GFP*
SH/PrP*	mPrP and EGFP	SH-SY5Y	SB/PrP*
SH/PrPΔCR*	mPrPΔ(105–125) and EGFP	SH-SY5Y	SB/ΔCR*
SH/mCherry*	mCherry	SH-SY5Y	LV/mCh*
SH/Sho*	mSho and mCherry	SH-SY5Y	LV/Sho(R)*
SH/Sho + PrP*	mPrP and EGFP	SH/Sho	LV/PrP(G)*
SH/ShoΔ(RXXX)_8_	mShoΔ(25–61) and mCherry	SH-SY5Y	LV/ShoΔ^(RXXX)8^(R)
SH/Sho(QXXX)_8_	mSho(QXXX)_8_ and mCherry	SH-SY5Y	LV/Sho^(QXXX)8^(R)
SH/ShoΔHD	mShoΔ(62–77) and mCherry	SH-SY5Y	LV/ShoΔ^HD^(R)
SH/Sho(PrPHD)	Sho(1–61)-PrP(111–133)-Sho(78–147) and mCherry	SH-SY5Y	LV/Sho^(PrPHD)^(R)
HEK/mCherry*	mCherry	HEK293	LV/mCh*
HEK/Sho*	mSho and mCherry	HEK293	LV/Sho(R)*
HEK/Sho + GFP*	EGFP	HEK/Sho	LV/GFP*
HEK/Sho + PrP*	mPrP and EGFP	HEK/Sho	LV/PrP(G)*

Cell cultures and plasmid vectors marked with asterisks are described in ref. 26.
