# Supplementary material for: The prion protein family member Shadoo induces spontaneous ionic currents in cultured cells
Source: Sci Rep. 2016 Nov 7;6:36441. doi: 10.1038/srep36441 (PMC5098206; doi:10.1038/srep36441)

The prion protein family member Shadoo induces spontaneous ionic currents in cultured cells

Antal Nyeste[1,2,#], Claudia Stincardini[3,#], Petra Bencsura[2], Milica Cerovic[4], Emiliano Biasini[3,4,*], Ervin Welker[1,2,*]

[1] Institute of Biochemistry, Biological Research Center, Hungarian Academy of Sciences, Szeged, Hungary

[2] Research Centre for Natural Sciences, Hungarian Academy of Sciences, Budapest, Hungary

[3] Dulbecco Telethon Laboratory of Prions and Amyloids, Center for Integrative Biology (CIBIO), University of Trento, 38123 Trento, ITALY

[4] Department of Neuroscience, Istituto di Ricerche Farmacologiche Mario Negri, 20156 Milano, ITALY

[#] These authors contributed equally to this work.

* To whom correspondence should be addressed:

Ervin Welker, Institute of Biochemistry, Biological Research Centre, HAS, Temesvari krt 62, H-6726, Szeged, Hungary. Tel: +36-62-599631, email: welker.ervin@brc.mta.hu

Emiliano Biasini, Dulbecco Telethon Laboratory of Prions and Amyloids, Center for Integrative Biology (CIBIO), University of Trento, Via Sommarive 9, 38123 Trento (TN), Italy. Tel: +39-0461-282740, email: biasinie@gmail.com

Supplementary Table S1: the DNA oligos used in the cloning processes.

**Primer**	**Sequence**
ShoΔHD-fwd	TACGGCTCCTCTCTGCGCGGCCTTGCTACCGGCTCT
ShoΔHD-rev	AGAGCCGGTAGCAAGGCCGCGCAGAGAGGAGCCGTA
V-ΔHD-fwd	TTTCCCTCATGAACGTTATCCCCTGATTCTGTG
V-ΔHD-rev	AGCTCTGCTCTTCCTGATGTCCTCCTCCAGCCAGAGCC
PrPHDlin-fw	TCGCGTGGCAGGGGCTGCGGCAGCTGGGGCAGTAGTGGGGGG CCTTGGTGGCTACATGCTGGGGAGCGCCATG
PrPHDlin -rev	GGCCCATGGCGCTCCCCAGCATGTAGCCACCAAGTCCCCC CACTACTGCCCCAGCTGCCGCAGCCCCTGCCAC
V-CMV-PrP-fwd	TTTGCAGGCGCGCCCGATGTACGGGCCAGATATACG
Sho-HD5-rev	CTGCAGCGTCTCAGCGAAGAGAGGAGCCGTAGCG
Sho-HD3 fw	GGTGTCGTCTCGGGCCTTGCTACCGGCTCTGGC
V-ins-rev	GAGCTGACAGGTGGTGGCAATGCCCCAACC
CMV-in-fwd	GGGTGGAGTATTTACGGTAAACTGCCCACTTGG
V-CMV-PrP-rev	CACTATTGTACAGGGCCCTCTAGATGCATGCTCGAGC

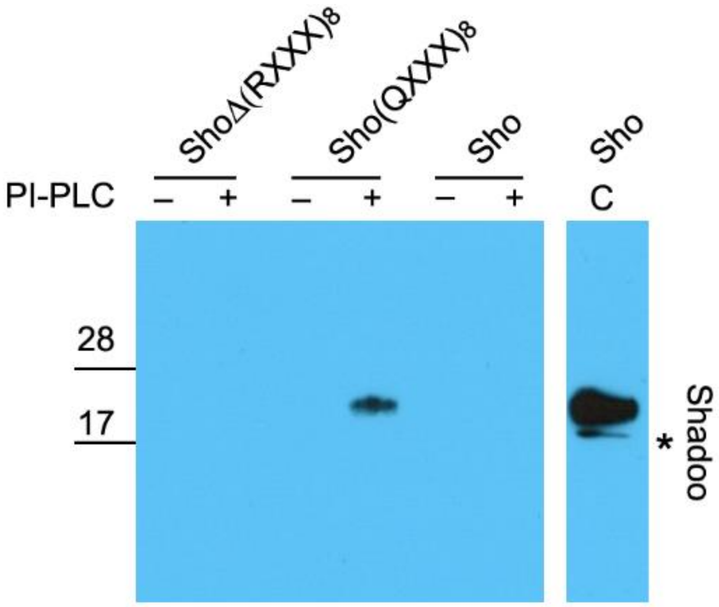

Supplementary Figure S2. Immunoblot of PI-PLC treated medium samples. Cell surface localization of Sho and its $(RXXX)_8$ domain mutant variants assessed by western blot analysis. TCA precipitated total protein samples from media supernatants of Sho, $Sho\Delta(RXXX)_8$ and $Sho(QXXX)_8$ expressing SH-SY5Y cells with (+) or without (-) PI-PLC-treatment were immunoblotted. A total cell lysate (C) of Sho overexpressing SH-SY5Y cells were also loaded (upper right panel) as control. *: marks nonspecific bands. Numbers and marks on the left indicate the positions of the corresponding molecular-weight size markers in kDa.

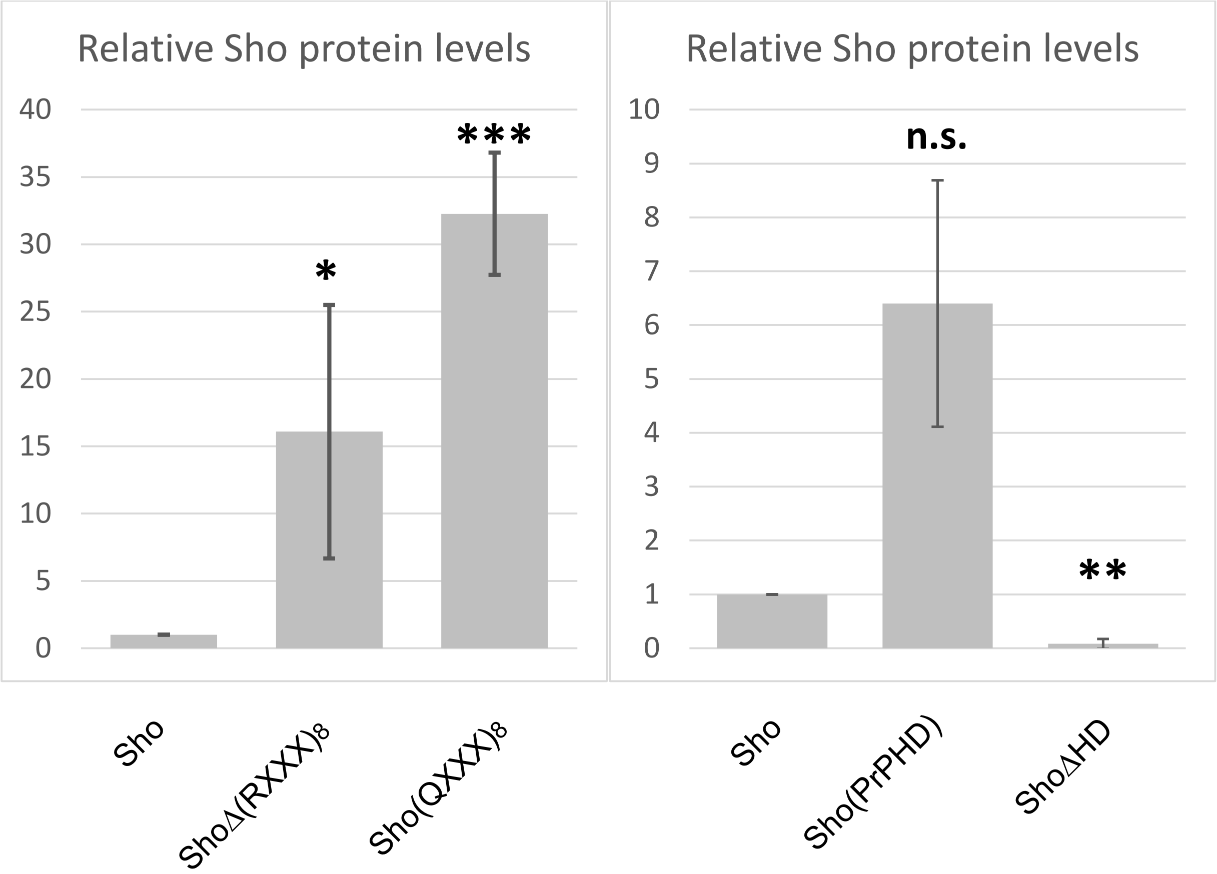

Supplementary Figure S3. Densitometric analysis of expression levels of various Sho proteins assessed on immunoblots of total cell lysates. Measured Shadoo band densities were normalized to the respective β-actin bands and the indicated Shadoo mutants were compared to the WT Shadoo. Bars show the means ± S.D. measured on n=3 independent western blot experiments. *: $p<0.05$, **: $p<0.01$, ***: $p<0.001$, n.s.: no significant difference ($p>0.05$) found, when compared to the mean of the wild-type Sho.

Supplementary Figure S4: Uncropped versions of the pictures of Western blot films shown on Figure 2 and 3.

Figure 2

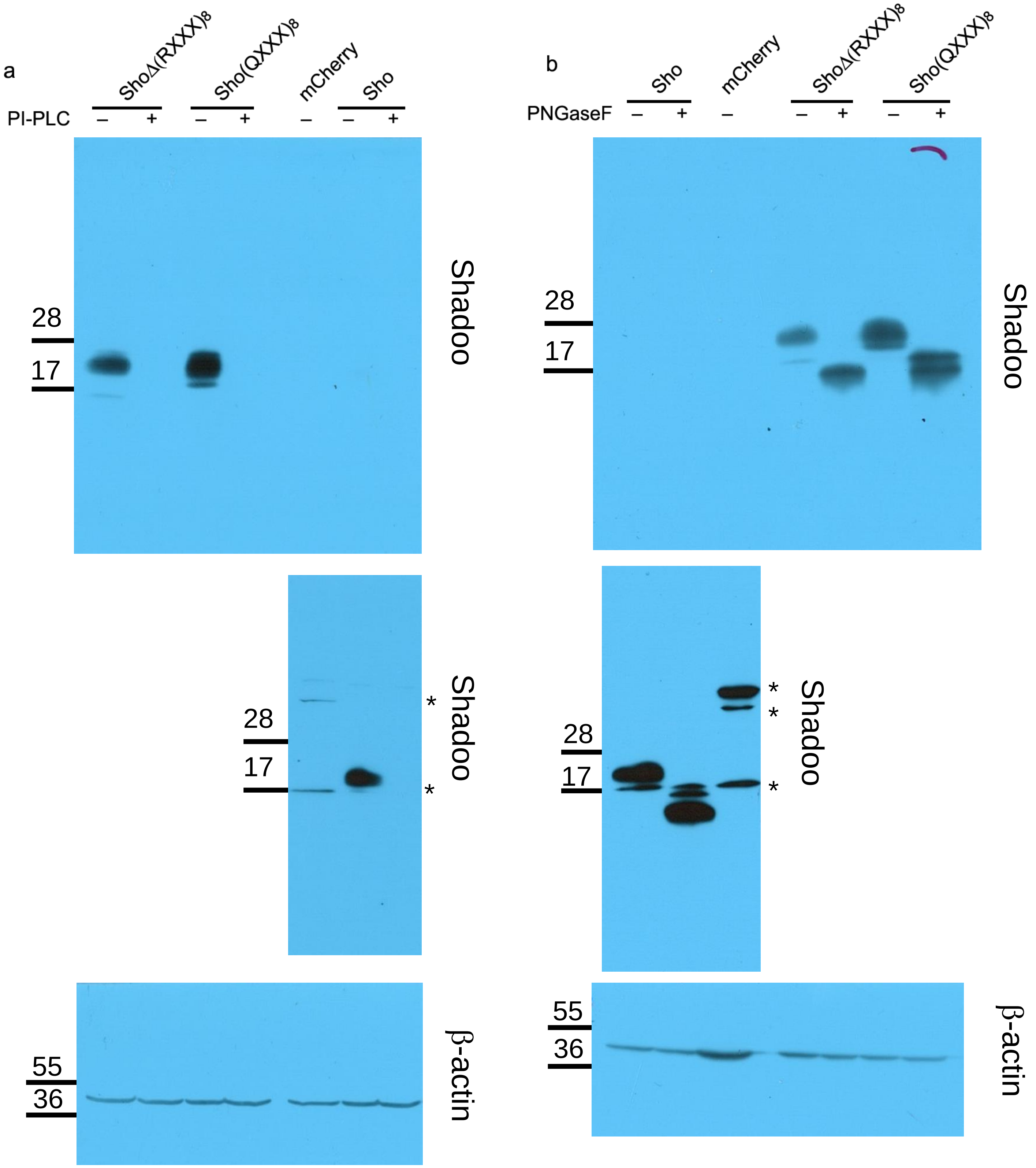

Supplementary Figure S4: uncropped western blot photos (cont.)

Figure 3

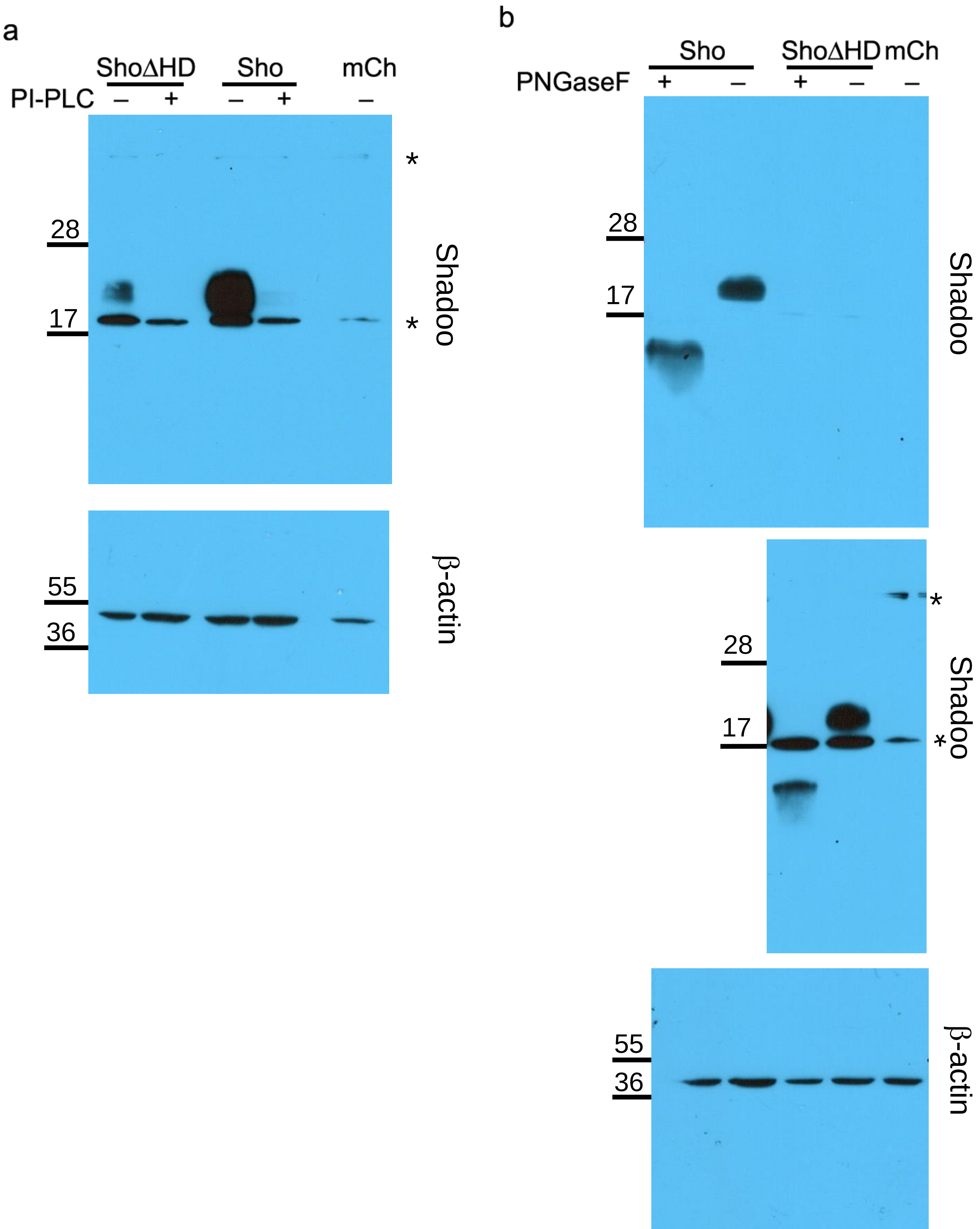

Supplementary Figure S4: uncropped western blot photos (cont.)

Figure 3

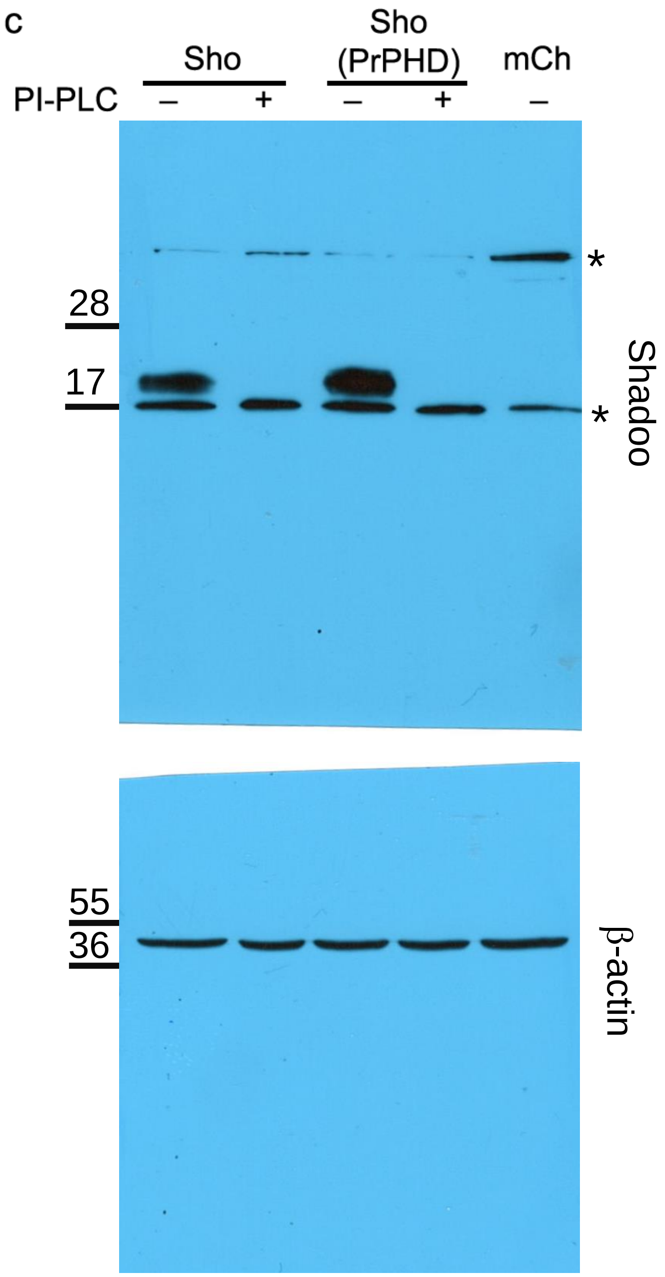

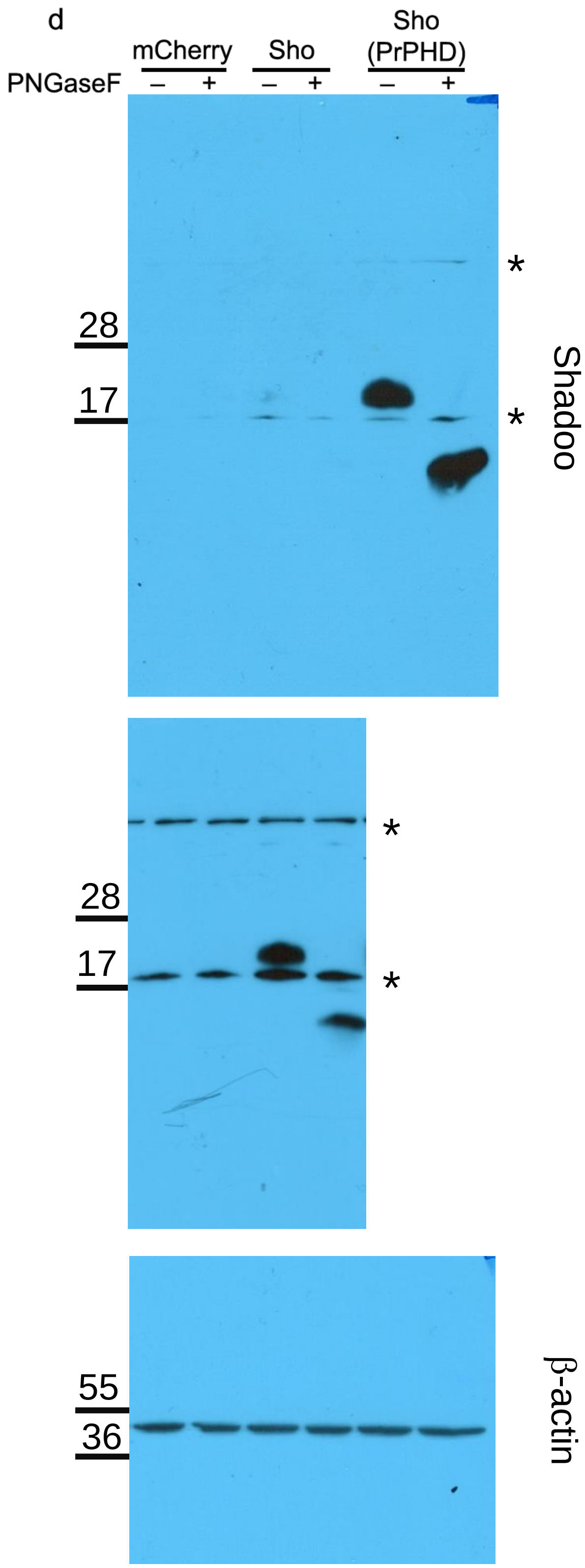

Supplement: Supplementary Information [file srep36441-s1.pdf]
